# Supplementary material for: β-Catenin Elicits Drp1-Mediated Mitochondrial Fission Activating the Pro-Apoptotic Caspase-1/IL-1β Signalosome in Aeromonas hydrophila-Infected Zebrafish Macrophages
Source: Cells. 2023 May 30;12(11):1509. doi: 10.3390/cells12111509 (PMC10252323; doi:10.3390/cells12111509)
Supplement: Supplementary file 1 [file cells-12-01509-s001.zip › cells-1984098-supplementary.pdf]

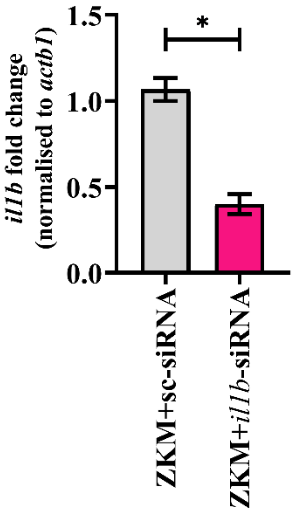

Figure S1. Transfection of *il1b* siRNA attenuates *il1b* expression in ZKM. ZKM transfected with sc-siRNA and *il1b* siRNA and *il1b* expression was studied using RT-qPCR. Vertical bars represent mean ± SEM (n=3). Asterisk represents significant difference between indicated groups (**p*<0.05).

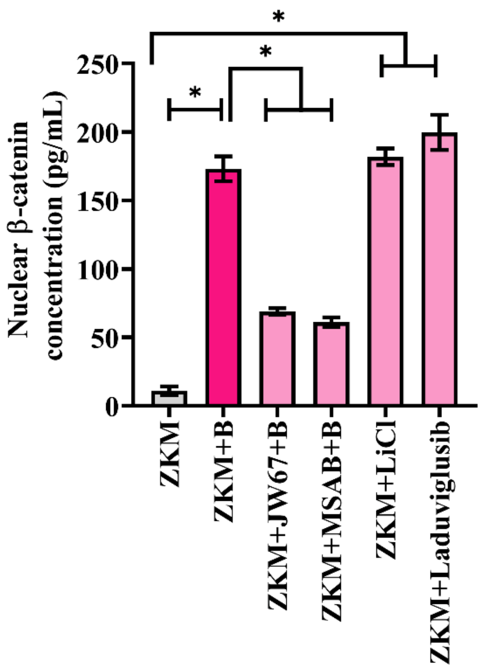

Figure S2. Effect of JW67, MSAB, LiCl and Laduviglusib on the nuclear translocation of β-catenin in ZKM. ZKM pre-treated with or without β-catenin inhibitors (JW67 and MSAB) for 1 h were infected with *A. hydrophila* and at 6 h p.i., nuclear β-catenin levels were measured using CTNNβ1 ELISA Kit. Similarly, ZKM were pre-treated with LiCl and Laduviglusib for 1 h and at 6 h p.i., nuclear β-catenin levels were measured. Vertical bars denote mean ± SEM (n=3). Asterisk (*) denotes significant difference between indicated groups (**p*<0.05). "+ B" mentioned in X axis represents "+ *A. hydrophila*".

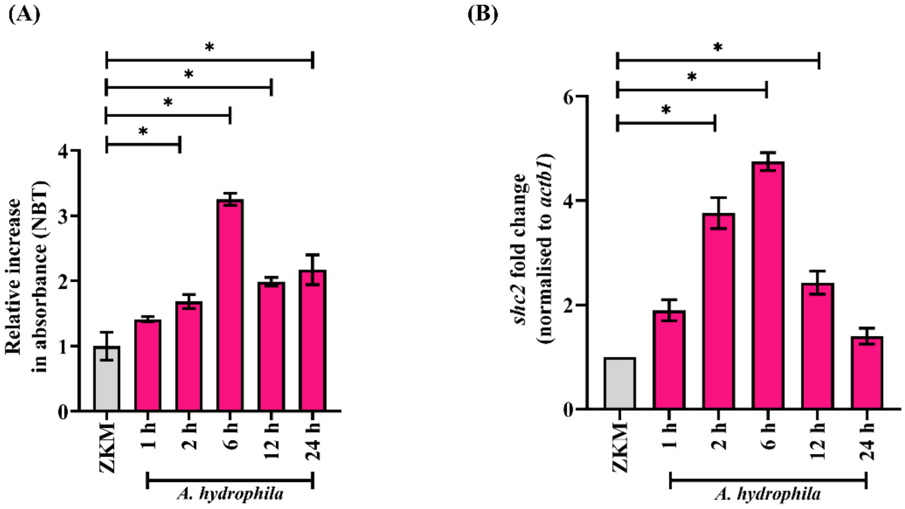

Figure S3. *A. hydrophila*-infected ZKM shows enhanced ROS generation and *shc2* mRNA expression. ZKM were infected with *A. hydrophila* and (A) superoxide generation was recorded at indicated time points p.i. using NBT assay, and (B) *shc2* mRNA expression was analysed by RT-qPCR at indicated time points p.i. Vertical bars denote mean ± SEM (n=3). Asterisk (*) denotes significant difference between indicated groups (*$p<0.05$).

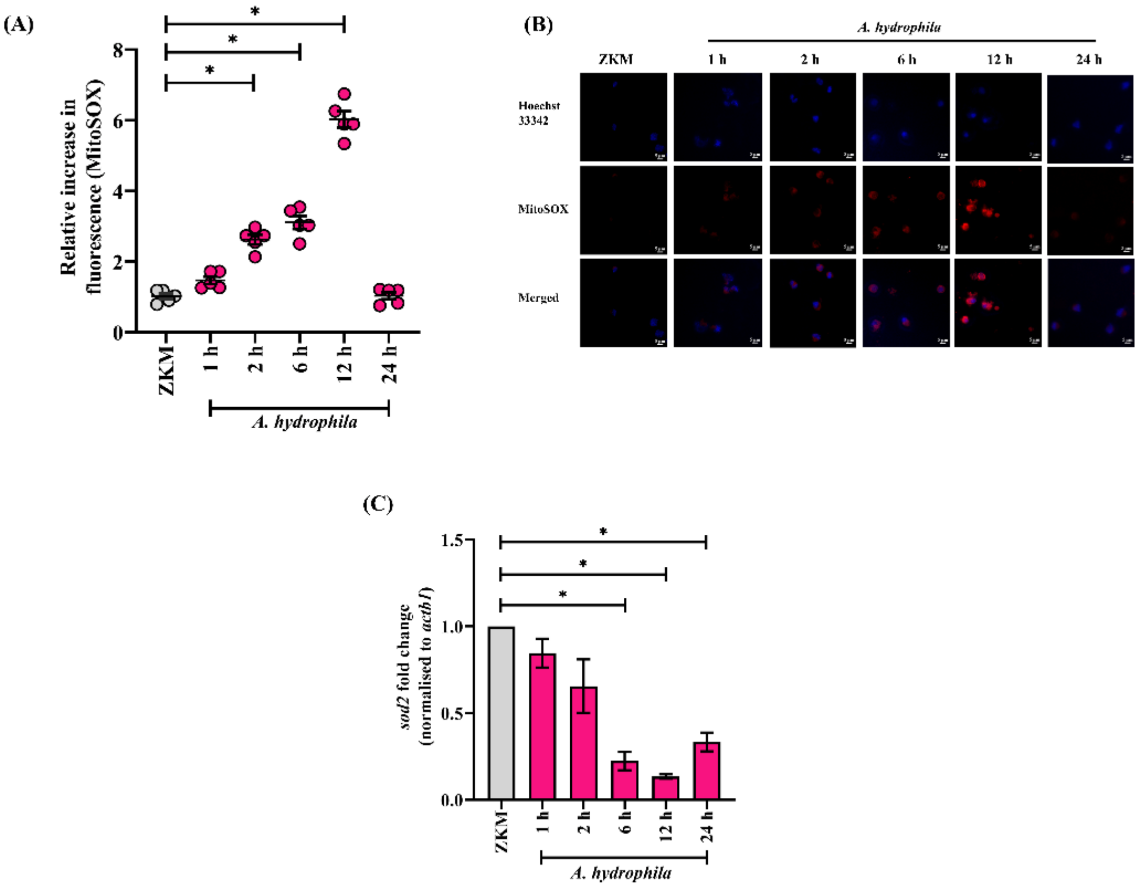

Figure S4. *A. hydrophila* infection induces mtROS production and downregulates *sod2* mRNA expression in ZKM. ZKM were infected with *A. hydrophila* and at indicated time points p.i., mtROS production was studied using MitoSOX (A) by fluorimeter, and (B) using fluorescence microscope. Fluorescence microscopic data is representative of three independent experiments (Scale- 5 µm). (C) ZKM were infected with *A. hydrophila* and at

indicated time points p.i., *sod2* mRNA expression were analysed using RT-qPCR. Vertical bars denote mean ± SEM (n=3). Asterisk (*) denotes significant difference between indicated groups (*p<0.05).

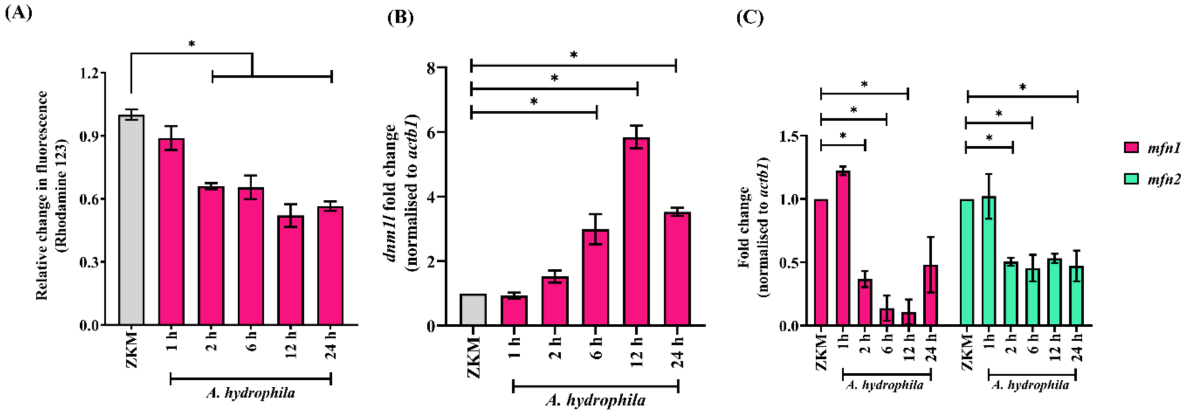

Figure S5. *A. hydrophila* infection induces $\Delta\Psi_m$ loss, upregulates *dnm1l* mRNA expression and downregulates *mfn1* and *mfn2* mRNA expression in ZKM. ZKM were infected with *A. hydrophila* and at indicated time points p.i., (A) $\Delta\Psi_m$ change was studied using Rhodamine 123, (B) *dnm1l* and (C) *mfn1* and *mfn2* mRNA expression were analysed using RT-qPCR. Vertical bars denote mean ± SEM (n=3). Asterisk (*) denotes significant difference between indicated groups (*p<0.05).

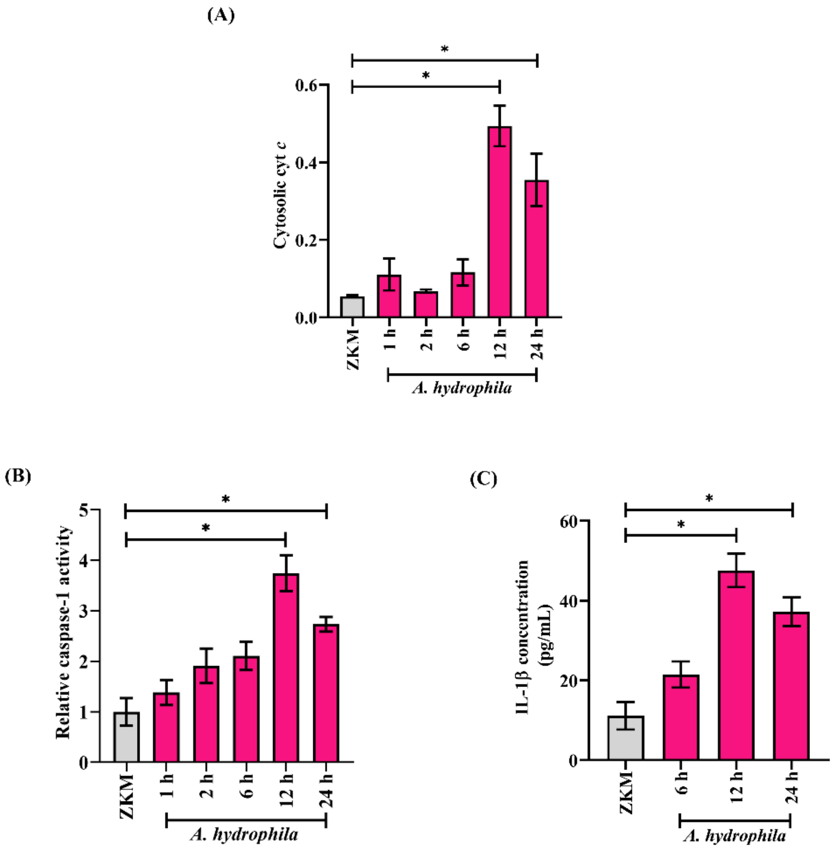

Figure S6. *A. hydrophila* infection induces cyt *c* release into cytosol and upregulates caspase-1 activity and IL-1β production in ZKM. ZKM were infected with *A. hydrophila* and (A) cytosolic cyt *c* levels were measured at indicated time points p.i., (B) relative changes in caspase-1 activity were measured at indicated time points p.i., and (C) IL-1β concentrations were measured at indicated time points p.i. Vertical bars represent mean ± SEM (n=3). Asterisk (*) denotes significant difference between indicated groups (*p<0.05).
